# Multiple cardiovascular risk factors in adolescents from a middle-income country: Prevalence and associated factors

**DOI:** 10.1371/journal.pone.0200075

**Published:** 2018-07-05

**Authors:** Thiago Veiga Jardim, Thomas A. Gaziano, Flávia Miquetichuc Nascente, Carolina de Souza Carneiro, Polyana Morais, Vanessa Roriz, Karla Lorena Mendonça, Thaís Inácio Rolim Póvoa, Weimar Kunz Sebba Barroso, Ana Luiza Lima Sousa, Paulo César Brandão Veiga Jardim

**Affiliations:** 1 Hypertension League–Federal University of Goias, S/N.–Setor Universitário, Goiânia, GO, Brazil; 2 Brigham & Women's Hospital—Division of Cardiovascular Medicine, Boston, Massachusetts, United States of America; 3 Harvard TH Chan School of Public Health—Department of Health Policy and Management, Center for Health Decision Science, Boston, MA, United States of America; 4 School of Physical Education and Therapy (ESEFFEGO)—State University of Goiás (UEG), Goiânia, GO, Brazil; Medical University Innsbruck, AUSTRIA

## Abstract

Multiple cardiovascular risk factors are directly related to the severity of atherosclerosis, even in children and adolescents. In this context accurate assessment of risk factors at the individual level play a decisive role in cardiovascular disease (CVD) prevention. The objective of this study was to estimate the prevalence of cardiovascular risk factors, the frequency of their coexistence in individuals, and identify possible determinants associated with this coexistence in Brazilian adolescents. A cross-sectional study with 1170 students (12–17 years) from public and private schools of a large city was conducted. In addition to family history, modifiable cardiovascular risk factors were assessed including: tobacco use, alcohol consumption, sedentary lifestyle, overweight/obesity, increased waist circumference, and high blood pressure (office and home). We built a linear regression model to identify determinants associated with increasing number of modifiable risk factors. Mean study population age was 14.7±1.6 years, 67% were enrolled in public schools and 33% in private ones. The majority of the adolescents had at least two risk factors (68.9%), more than 10% had more than 4 risk factors, and in only 6.7% of the sample no risk factor was identified. Family history of CVD (β-coefficient = 1.20; 95%CI 1.07–1.34; p<0.001), increasing age (β-coefficient = 0.08; 95%CI 0.04–0.11; p<0.001), and being enrolled in private schools (β-coefficient = 0.16; 95%CI 0.02–0.30; p = 0.023) were directly associated with the modifiable CV risk factors. In conclusion, the prevalence of multiple cardiovascular risk factors was high in the population of adolescents studied. School based interventions should be addressed to change this scenario.

## Introduction

Cardiovascular diseases (CVD) are the leading cause of death worldwide and the burden of these diseases falls mainly on low and middle-income countries[1]. Projections show that deaths due to CVD will increase to approximately 23 million by 2030[2], when CVD will remain the leading cause of mortality.

Cardiovascular risk factors such as hypertension, dyslipidemia, diabetes, smoking, obesity, sedentary lifestyle and family history of CVD, are the underlying factors for most cardiovascular events[3]. It is well known that controlling classical modifiable risk factors can significantly decrease the prevalence of CVD therefore reducing its attributed mortality[4]. Unfortunately changes in childhood and adolescents habits are contributing to increase the rates of all traditional CVD risk factors[5].

Adolescence is a transition stage between childhood and adulthood and is an important phase of development of future lifestyle behaviours[6]. Cardiovascular risk factor prevalence in youth and adult life are highly correlated[7]. Further, there is a positive relation between number of cardiovascular risk factors and severity of atherosclerosis, even in children and young adults[8]. Therefore, a better comprehension of both presence and number of multiple risk factors in young people can significantly contribute to actions that may modify their natural history and prevent CVD onset[7].

The prevalence of cardiovascular risk factors in adolescents has been studied in middle-income countries[9–12]. However no published data, to our knowledge, has assessed neither the multiple prevalence of these factors nor its related factors. Accordingly, this study aimed to estimate the prevalence and associated factors of cardiovascular risk factors among adolescents enrolled in public and private schools of a large city of the Midwest region of Brazil, with a particularly interest in the prevalence of multiple risk factors. The study rationale was that if modifiable cardiovascular risk factors could be identified in early stages of life, then potential public health policy and/or school-based interventions could be developed to reduce this increasingly common situation among adolescents.

## Materials and methods

This school-based cross-sectional study was conducted with secondary school students (12–17 years) from both public and private schools in the city of Goiânia, within the state of Goiás, Brazil, in 2011. Goiânia has a population of 1,302,001 inhabitants and is located in the Midwest region of Brazil; its land area is 732.80 km^2^, and its population density is 1776.75 hab/km^2^ [13].

The study was approved by the Research Ethics Committee of the Institution coordinating the project (Clinics Hospital–Federal University of Goias, protocol n° 017/2010). Both eligible adolescents, who agreed to participate in the study, and their guardians signed an informed consent form.

Sample size was calculated for a main epidemiological project that aimed to assess white coat and masked hypertension in adolescents and its correlations with left ventricular mass index and insulin resistance[9]. This calculation considered a population of 133,528 students enrolled both in public and private schools of Goiânia[13], masked hypertension prevalence of 7%, white-coat hypertension prevalence of 10%, an absolute error of 3% and a confidence level of 95%. These parameters determined a sample size of 1059 students. A total of 1170 students were included, approximately 10% more than the estimated number, to cover eventual losses.

A total of 132 public and private schools from the nine regions of the city were identified and the ratio of two public schools for each private one was established. After that, six public and three private schools from each region were drawn and then invited to participate. The school contact followed the drawing order. If a school did not agree to participate, the replacement was made by the same school type (public replaced by public and private replaced by private). At the end, 26 schools agreed to participate (17 public and nine private). Adolescents’ selection was performed by random drawing of the students enrolled in the institutions, stratified by age and sex[14].

The exclusion criteria were: physical disabilities that prevented the practice of physical activities (PA); pregnancy; disabling chronic diseases and use of continuous medication.

Data was collected by a trained team over 13 months. The team consisted of four supervisors and nine data collectors (7 interviewers and 2 anthropometrists) who were students from Physical Therapy and Nutrition programs and had been previously trained.

This study used a standardized questionnaire that included the International Physical Activity Questionnaire (IPAQ)[15], anthropometric measurements (height, weight and waist circumference) and blood pressure (BP) measurements. This questionnaire provided information about school type (public or private); adolescent identification (name, date of birth, age, sex, race/ethnicity: white or non-white, telephone number and address); personal history (current diseases and medications); family history of cardiovascular disease (any history of angina, acute myocardial infarction, stroke, or necessity of revascularization in parents or grandparents); tobacco use, alcohol consumption and dietary habits.

Office measurements were performed by trained health professionals, based on the Brazilian Hypertension Guidelines[16]. The procedure took place at the schools, on two different days (one-week interval) and with two measurements (with a three-minute interval) at each time point. For the analysis, the mean of the second set of measurements was used since it has shown a better correlation with home BP values in this population[17]. We utilized a semi-automatic equipment (OMRON, model HEM-705CP), which was validated to be used in adolescents[18]. Cuffs in three different sizes (9 x 16 cm, 13 x 23 cm and 15 x 30 cm) were selected according to the adolescent’s right arm circumference (80 to 100%).

The same equipment, cuffs, and techniques used for the office measurements were also used for home blood pressure monitoring (HBPM). Adolescents received the device at school and were told to perform two measurements (with three-minute intervals) during the day (between 06:00 and 10:00 a.m.) and two at night (between 06:00 and 10:00 p.m.) over 6 days, for a total of 24 readings. Exams were consider valid if at least 50% (12 readings) were performed[19]. The overall mean value was used for this analysis.

Hypertension was diagnosed in those adolescents who presented office and/or home BP measurements (systolic and/or diastolic) at or above the 95^th^ percentile for the corresponding age, gender and height[14, 16], or those whit self-reported hypertension.

The nutritional status of adolescents was assessed using body mass index (BMI = weight [kg] / height [m]^2^) which was classified according to the specific reference standard for the age and sex proposed by the World Health Organization (WHO) (2007)[20].

The cut-off points for waist circumference (WC) were adjusted by age and sex according to international classifications[21], since there are no Brazilian data establishing the 90^th^ percentile, adjusted for sex, as an indicator of metabolic changes.

The short-form (Version 8) of the IPAQ was applied to estimate the prevalence of sedentary lifestyle, considering the PA practiced in the previous week as a reference[15]. The questions addressed the frequencies and durations of light (walking), moderate, and vigorous PA. Sedentary lifestyle was defined as less than 300 minutes of moderate or vigorous physical activity per week[22].

Regarding alcohol consumption adolescents were asked if they had any alcohol consumption in the last 30 days and the positive answers were defined as alcohol consumption. Smoking was assessed by self-report of having smoked at least one day over the previous 30 days.

Modifiable CV risk factors were defined as: hypertension, overweight (BMI ≥ 85^th^ percentile for a given age and sex), increased WC (≥90^th^ percentile for age and sex), smoking, alcohol consumption and sedentary lifestyle. Non-modifiable risk factor was defined as family history of CVD.

Data were entered in duplicate in Epiinfo 3.5.1 software. Variables were analysed using the software Stata 14.0. Population descriptive analysis with mean values and standard deviation of continuous variables was performed. Pearson’s Chi-square Test was used to evaluate the differences between sexes. Cardiovascular risk factors prevalence was calculated with their 95% confidence interval. Comparison between proportions was performed with a two-sample proportion test. A linear regression model was built to identify factors associated with the number of existing modifiable cardiovascular risk factors. The predictors included in this model were sex, age, school type, race/ethnicity and family history of cardiovascular disease. The results are presented using β-coefficients and 95% confidence intervals. A significance level was set as 5%.

## Results

A total of 1170 students enrolled in public (67%) and private (33%) schools were evaluated. The mean age of the population was 14.7 (±1.6) years with no differences between sexes. No BMI difference was observed between sexes as well. Mean office blood pressure was 112.3±12.7 x 66.5±8.1 mmHg and mean home blood pressure was 112.9±10.3 x 66.6±6.6 mmHg, and both of them were higher among males. Clinical characteristics of these participants are summarized in Table 1.

**Table 1:** Population overall characteristics with sex stratification.

Indicator	Overall		Male		Female		
	*n*	Mean (±SD)	*n*	Mean (±SD)	*n*	Mean (±SD)	p-value*
**Age (years)**	1,170	14.7±1.6	549	14.7±1.6	621	14.7±1.6	0.691
**Weight (kg)**	1,169	57.3±13.4	548	61.2±14.4	621	53.8±11.3	<0.001
**Height (m)**	1,169	1.65±0.91	548	1.70±0.94	621	1.61±0.66	<0.001
**Body Mass Index (kg/m**^**2**^**)**	1,169	20.7±3.8	548	21.0±3.8	621	20.5±3.7	0.062
**Waist Circumference (cm)**	1,168	70.8±9.1	547	72.9±9.4	621	69.0±8.3	<0.001
**Mean office SBP**^**a**^ **(mmHg)**	1,023	112.3±12.7	487	117.5±13.1	536	107.7±10.4	<0.001
**Mean office DBP**^**b**^ **(mmHg)**	1,023	66.5±8.1	487	67.2±8.4	536	65.9±7.8	0.013
**Mean home SBP**^**c**^ **(mmHg)**	1025	112.9±10.3	487	116.9±10.1	538	109.2±9.1	<0.001
**Mean home DBP**^**d**^ **(mmHg)**	1025	66.6±6.6	487	67.3±6.9	538	66.0±6.3	0.003
**Heart rate (bpm)**	1,161	78.9±11.8	541	76.2±11.5	620	81.3±11.6	<0.001
**Birth weight (g)**	826	3275.9±596.0	356	3331.0±626.0	470	3234.2±569.4	0.020

a. Mean systolic blood pressure values of second measurements in different moments (one week apart)

b. Mean diastolic blood pressure values of second measurements in different moments (one week apart)

c. Mean systolic blood pressure values of all home blood pressure measurements.

d. Mean systolic blood pressure values of all home blood pressure measurements.

*Difference between male and female—Statistically significant at α = 0.05

Cardiovascular risk factors prevalence assessed in this group of adolescents is presented in Table 2. Sedentary lifestyle prevalence was extremely high in the overall population and female adolescents were more likely sedentary than males. Alcohol consumption was also highly prevalent and more common in female sex. On the other hand, hypertension and overweight were more prevalent in male adolescents.

**Table 2:** Prevalence of cardiovascular risk factors in the overall study population and stratified by sex.

	Overall		Male		Female		
	*n*	n (%)	*n*	n (%)	*n*	n (%)	p-value*
**Hypertension**^**a**^	1,025	**118 (11.5)**	492	**76 (15.4)**	533	**42 (7.9)**	<0.001
**Overweight**^**b**^	1,169	**272 (23.3)**	548	**159 (29.0)**	621	**113 (18.2)**	<0.001
**Increased WC**^**c**^	1,168	**40 (3.4)**	547	**19 (3.5)**	621	**21 (3.4)**	0.931
**Smoking**^**d**^	1,170	**15 (1.3)**	549	**8 (1.4)**	621	**7 (1.1)**	0.617
**Alcohol consumption**^**e**^	1,170	**730 (62.4)**	549	**323 (58.8)**	621	**408 (65.5)**	0.018
**Sedentary lifestyle**^**f**^	1,165	**822 (70.6)**	548	**326 (59.5)**	617	**496 (80.4)**	<0.001
**CVD family history**^**g**^	1,170	**423 (36.1)**	549	**186 (33.9)**	621	**237 (38.2)**	0.128

a. Office blood pressure ≥ 95^th^ percentile or home blood pressure ≥ 95^th^ percentile.

b. Body mass index ≥ 85^th^ percentile.

c. Waist circumference ≥ 90^th^ percentile.

d. Smoked at least one day over the previous 30 days.

e. Alcohol consumption in the last 30 days.

f. Less than 300 minutes of moderate or vigorous physical activity in the last week.

g. Cardiovascular disease in parents or grandparents.

*Difference between male and female—Statistically significant at α = 0.05

When the prevalence of the modifiable risk factors was compared between public and private schools the rates of overweight were higher in public schools while sedentary lifestyle was more common in private schools. There were no other significant differences between the groups with regards to the remaining modifiable risk factors, as shown in Table 3.

**Table 3:** Prevalence of modifiable risk factors by public and private schools.

	Public Schools		Private Schools		
	%	95% CI	%	95% CI	p-value*
**Hypertension**^**a**^	**12.6**	9.9–15.3	**10.9**	7.2–14.5	0.459
**Overweight**^**b**^	**31.4**	26.8–36.1	**19.3**	16.5–22.0	<0.001
**Increased WC**^**c**^	**2.8**	1.6–4.0	**4.7**	2.6–6.8	0.100
**Smoking**^**d**^	**1.5**	0.7–2.4	**0.7**	0.09–1.6	0.283
**Alcohol consumption**^**e**^	**60.8**	57.4–64.2	**65.4**	60.7–70.2	0.126
**Sedentary lifestyle**^**f**^	**68.6**	65.3–71.8	**74.7**	70.4–79.1	0.030

a. Office blood pressure ≥ 95^th^ percentile or home blood pressure ≥ 95^th^ percentile.

b. Body mass index ≥ 85^th^ percentile.

c. Waist circumference ≥ 90^th^ percentile.

d. Smoked at least one day over the previous 30 days.

e. Alcohol consumption in the last 30 days.

f. Less than 300 minutes of moderate or vigorous physical activity in the last week.

*Statistically significant at α = 0.05

**Fig 1:**
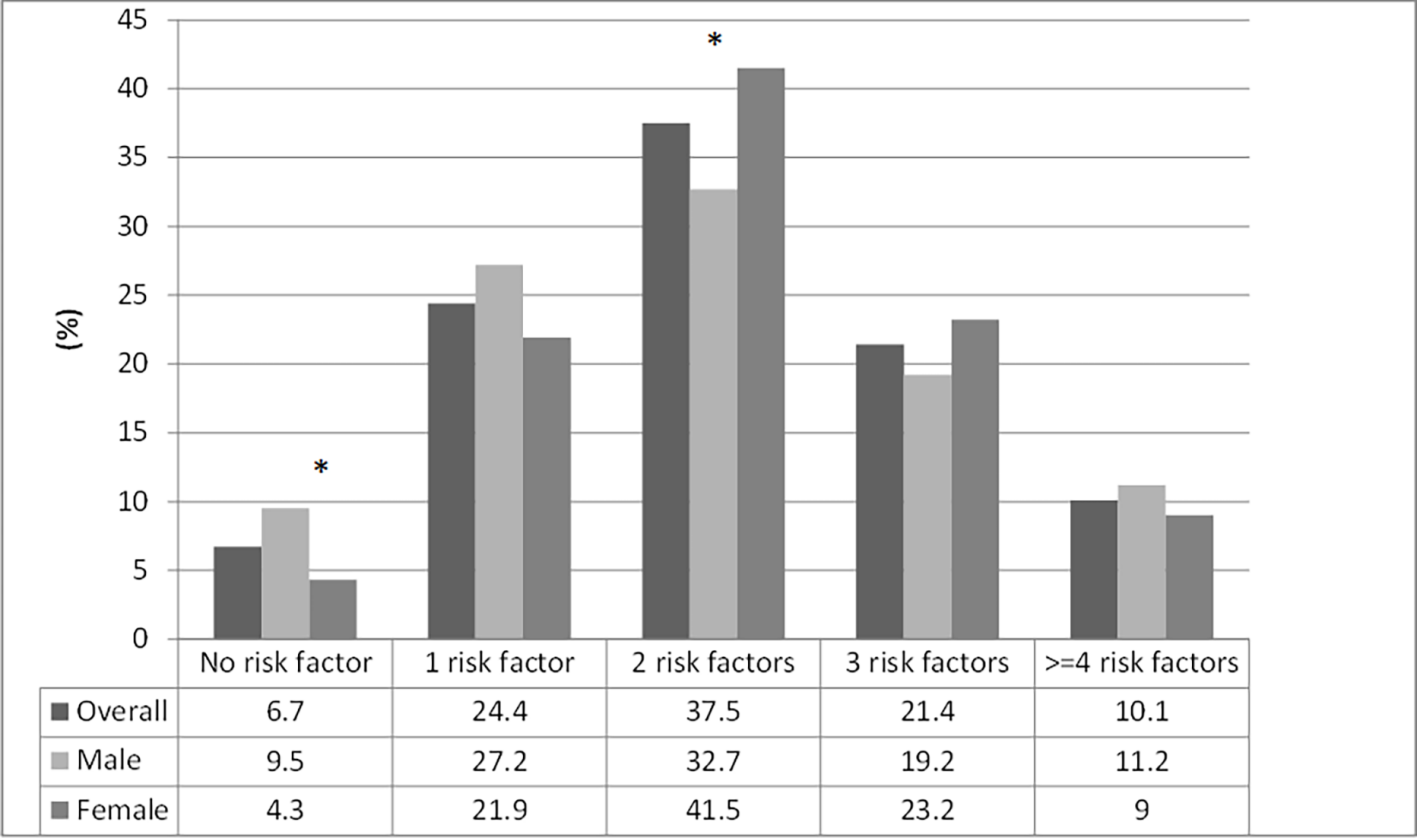
Sample distribution by number of cardiovascular risk factors^a^ (n = 1025). **a.** Hypertension—Office blood pressure ≥ 95^th^ percentile or home blood pressure ≥ 95^th^ percentile, overweight—Body mass index ≥ 85^th^ percentile; increased waist circumference—waist circumference ≥ 90^th^ percentile; smoking—smoked at least one day over the previous 30 day; alcohol consumption–alcohol consumption in the last 30 days; sedentary lifestyle—less than 300 minutes of moderate or vigorous physical activity in the last week; family history of cardiovascular disease. *Difference between male and female—Statistically significant at α = 0.05.

Most adolescents had at least two risk factors (68.9%) and more than ten percent had more than 4 risk factors. In only 6.7% of the sample no cardiovascular risk factor was identified (Fig 1).

Family history of CVD, increasing age, and being enrolled in private schools were directly associated with the number of modifiable CV risk factors, as shown in Table 4.

**Table 4:** Linear regression coefficients for the number of existing modifiable cardiovascular risk factors.

Variables	*β-*coefficient	[95% CI]	p-value*
**CVD family history**^**a**^	1.20	1.07–1.34	<0.001
**Age**	0.08	0.04–0.11	<0.001
**Private school**	0.16	0.02–0.30	0.023
**Male sex**	-0.10	-0.22–0.03	0.131
**Race/ethnicity (White)**	-0.07	-0.20–0.05	0.272

a. Cardiovascular disease in parents or grandparents.

*Statistically significant at α = 0.05

## Discussion

This study was conducted in the Midwest region of Brazil with a representative sample of students aged 12–17 years from public and private schools assessing the prevalence of multiple cardiovascular risk factors and its related factors. The overall prevalence of some cardiovascular risk factors such as alcohol consumption, sedentary lifestyle, family history of cardiovascular disease, overweight and hypertension was high considering such a young population. Even more concerning was the assessment of multiple cardiovascular risk factors in which most of the adolescents had at least two risk factors (68.9%) and more than 10% had more than four. Adolescents from public schools, older and with a family history of CVD were more likely to have a higher number of modifiable CV risk factors.

The sample characteristics, described in table 1, were very similar to the overall characteristics of adolescents living in this part of the country, and even in other parts like the South and Southwest regions[12, 23]. As shown in previously epidemiological studies[12] and population surveys[23], at this age group the mean weight, height and waist circumference of males is higher than females. These differences were not seen in BMI mean values. Another point leading to the same conclusion is the mean age of the population (14.7 years). It was exactly the same as a national school-based study conducted in Brazilian adolescents[24]. It suggests that the sampling methodology was adequate and should be consider in future school-based studies.

The prevalence of the seven cardiovascular risk factors assessed was very distinct. The most prevalent conditions were sedentary lifestyle, alcohol consumption, family history of cardiovascular disease and overweight. Since family history of cardiovascular disease is a non-modifiable risk factor and there is not much to be done to change its high prevalence, this result must be seen as an alert, since it increases the cumulative risk of these adolescents.

High prevalence of sedentary lifestyle must be addressed both locally and at the policy level. These high rates of physical inactivity at younger ages were found all over the world[25]. We found an even higher prevalence among females and students from private schools. This female pattern is frequent[26, 27] and interventions to change this reality in girls must be implemented at even younger ages.

Alcohol consumption may not be considered a traditional risk factor[28], even though reduction of alcohol consumption, even for light to moderate drinkers, is beneficial for cardiovascular health[29]. In adolescents, alcohol consumption is usually related to binge intake, which has been associated with higher levels of alcoholism in adulthood [6], a number of diseases and unsafe behaviours [30]. Our prevalence was high (62.4%), and higher than international (34.9%) [30] and national data (21–23%)[24, 31]. Maybe the definition used that considered any alcohol intake in the last 30 days can explain such difference. Nevertheless, raising the attention to such an important point as adolescents’ alcohol consumption can only be beneficial.

Overweight and increased waist circumference prevalence were 23.3% and 3.4% respectively, and were similar to recently published Brazilian data (25% and 3.3%, respectively)[12, 32]. If the prevalence of overweight was higher among male adolescents the opposite was seen for increased waist circumference. It can be explained by the age of our population and the different patterns of changes in waist circumference between boys and girls throughout time. Mean waist circumference increased with age in both sexes, but for girls, curves began to plateau after the age of 13 years whereas, for boys, waist percentile curves continued to increase more sharply after this age[33]. Another difference found was the higher prevalence of overweight in students from public schools. Considering the increased risk of cardiovascular abnormalities related to long-term exposure to excessive adiposity[34] and the strong correlation of overweight in younger ages with older ages[7] it is strongly recommended to implement interventions to change this reality even before adolescence with a particularly attention to students from public schools.

The prevalence of hypertension reported in this study (11.5%) is slightly higher than previously published data from Brazilian adolescents (8.1–9.6%) [32, 35] but similar to results from the same part of the country (Midwest region) (11.7%) [36]. When compared to data from different countries (11.2%)[37], the results are concordant. Another similarity is related to the higher prevalence in males when compared to females, which is repeatedly shown in similar studies[32, 35–37].

Currently smoking habit among the population of our study was very low (1.3%) and lower than the usually reported Brazilian adolescent’s prevalence (5.7% to 6.1%) [11, 38]. Maybe the methodology used in which adolescents needed to report to an interviewer their smoking habit can explain the difference, since at younger ages such a methodology can underestimate the real smoking prevalence[39].

When the cumulative number of risk factors was assessed the prevalence of subjects with at least two risk factors was extremely high among our population and even higher than results from high income countries[40, 41]. Even more concerning is that approximately 10% of the adolescents included had more than 4 risk factors in such young ages, which is also higher than the results from a high income country sample[41]. It is well known that the number of cardiovascular risk factors are directly associated to the severity of atherosclerosis, even in children and young adults, as shown by an autopsy-based study published by the Bogalusa Heart Study group in 1998[8]. Since our results indicate that adolescents from middle-income countries are being exposed to multiple cardiovascular risk factors this situation will lead, if nothing changes, to a high number of cardiovascular outcomes in the future, maintaining the high burden of CVD in the developing world into the next generation[1].

The inclusion of adolescents who were enrolled in schools, excluding those out of school is a potential limitation of this study, particularly in Brazil, where the rates of adolescents not attending school are high (approximately 9%)[13]. The same methodology was used in previously published studies from other parts of the world [6, 34], and can be justified by the easy access to adolescents enrolled in schools as well as the easier implementation of interventions in these populations.

From a health policy perspective our results must be seen as an alert and a call for changes in the way cardiovascular risk factors are handled. Although our linear regression model showed that family history of CVD, increasing age, and being enrolled in private schools were directly associated with the number of modifiable cardiovascular risk factors, interventions to decrease this number should be addressed to all adolescents. Effective school-based interventions are required for middle-income countries if we want to see positive and real changes in the global burden of cardiovascular diseases.

## Conclusion

The prevalence of multiple cardiovascular risk factors was high in the population of adolescents studied. In most of the sample at least two risk factors could be detected while more than 10% had four risk factors or more. Adolescents from public schools, older and with a family history of cardiovascular disease were more likely to have a higher number of modifiable CV risk factors. School based interventions should be addressed to change this scenario.

## Supporting information

S1 Dataset (XLSX)
